# The population structure and genetic diversity of *Listeria monocytogenes* ST9 strains based on genomic analysis

**DOI:** 10.3389/fmicb.2022.982220

**Published:** 2022-11-08

**Authors:** Zexuan Song, Shunshi Ji, Yan Wang, Lijuan Luo, Yiqian Wang, Pan Mao, Lingling Li, Huaying Jiang, Changyun Ye

**Affiliations:** ^1^State Key Laboratory of Infectious Disease Prevention and Control, National Institute for Communicable Disease Control and Prevention, Collaborative Innovation Center for Diagnosis and Treatment of Infectious Diseases, Chinese Center for Disease Control and Prevention, Beijing, China; ^2^School of Biotechnology and Biomolecular Sciences, University of New South Wales, Sydney, NSW, Australia; ^3^Department of Clinical Laboratory, People's Hospital of Xindu District, Chengdu, China

**Keywords:** *Listeria monocytogenes*, ST9, plasmid, prophage, Tn554-like, InlA

## Abstract

*Listeria monocytogenes* is a ubiquitous foodborne pathogen causing both invasive and non-invasive listeriosis. Sequence type (ST) 9 strains is common in food and food processing environments. In this study, the whole-genome sequences (WGS) of 207 ST9 isolates from different sources, geographical locations (14 countries), and isolated years were analyzed. The ST9 isolates were divided into three clusters after phylogenetic analysis; 67.63% of ST9 isolates contained putative plasmids with different sizes and genomic structure, the putative prophages inserted in the chromosome at ten hotspots, and seven types of premature stop codon (PMSC) mutations in *inlA* were found in 81.86% of the ST9 isolates. In addition, 78.26% of ST9 isolates harbored Tn554-like elements carrying arsenic resistance genes. All the ST9 isolates conservatively contained environment-resistance genes on the chromosome. This analysis of population structures and features of ST9 isolates was aimed to help develop effective strategies to control this prevalent pathogen in the food chain.

## Introduction

*Listeria monocytogenes* is a facultative intracellular pathogen causing listeriosis, a severe foodborne disease with a high fatality rate (20–30%). Invasive listeriosis is particularly dangerous for older adults, pregnant women, neonates, and immunocompromised people leading to bacteremia, neurolisteriosis, and maternal or neonatal infections (de Noordhout et al., 2014). Listeriosis is mostly linked to ready-to-eat (RTE) food like fresh produce and lightly processed meat products (Fagerlund et al., 2020). Recently, the largest outbreak of listeriosis with 1,060 confirmed cases and 216 cases of death was reported in South Africa and linked to RTE meat product contamination (Thomas et al., 2020).

*Listeria monocytogenes* can be divided into four evolutionary lineages (Orsi et al., 2011), four PCR serogroups, and 14 serotypes (Feng et al., 2020), and further subdivided into clonal complexes (CCs) and sequence types (STs) by multi-locus sequence typing (MLST) (Chenal-Francisque et al., 2011). The predominant isolates from food and human are distinct. CC1, CC2, CC4, and CC6 are infection-associated clones usually causing sporadic or outbreak listeriosis, while CC9 and CC121 are strongly food-associated clones that mostly infect immunocompromised individuals (Maury et al., 2016).

ST9 isolate is the most frequent subtype of CC9 in lineage II which is seen in many countries (Wang et al., 2012; Ebner et al., 2015; Cabal et al., 2019), and the human/food ratio of isolates is up to 0.5:1 (Chenal-Francisque et al., 2011). Furthermore, ST9 is a common subtype of *L. monocytogenes* that contaminates meat products and meat-processing environments (Stoller et al., 2019; Fagerlund et al., 2020), but is rare in other foods and food-processing environments, such as fruits, aquatic products, and raw milk products (Chen et al., 2018; Maury et al., 2019). ST9 isolates can remain in food-associated environments, especially in slaughterhouses and processing chains (Luo et al., 2017; Melero et al., 2019). Among 680 isolates from the two largest meat-processing plants in Norway, 70% belonged to ST9 (Møretrø et al., 2017).

Some genetic determinants could help *L. monocytogenes* isolates to survive and multiply in stress conditions. As the mobile genetic element, plasmid could benefit the host strain to survive and multiply under variable stress conditions (Naditz et al., 2019), increase the virulence capacity of the isolates (Kropac et al., 2019), and mediate antibiotic resistance by horizontal gene transfer (Guglielmetti et al., 2009). Prophages could accelerate the evolution of *L. monocytogenes* and provide host isolates the advantage to adapt and survive under stress condition (Wang et al., 2010; Verghese et al., 2011).

A longitudinal study revealed the transmission patterns of *L. monocytogenes* ST9 isolates by whole-genome sequencing (Fagerlund et al., 2020), but the molecular mechanism of the persistence of ST9 isolates in meat products is currently unknown. Genomic features of some sequence types of isolates such as ST121, ST204, ST87, and ST8 have been described (Schmitz-Esser et al., 2015; Fagerlund et al., 2016; Fox et al., 2016; Wang et al., 2019), but less is known about the ST9 isolates. Thus, we analyzed the population structure and genetic features of 207 isolates of ST9 *L. monocytogenes* from various sources, isolation times, and different regions of the world in this study.

## Materials and methods

### *L. monocytogenes* sequence type (ST) 9 isolates

The genome of a total of 207 ST9 isolates from various sources, diverse geographic locations, and isolation times was selected for in-depth analysis, including 68 newly sequenced genomes from China, and 139 publicly available genomes (13 complete and 126 draft genomes) from 13 other countries (Supplementary Table S1). There were 142, 31, 29, and 4 ST9 isolates from food, human, the environment, and animals respectively, with one isolate from an unknown source.

### Genome sequencing and annotation

Whole-genome sequencing of 68 ST9 isolates of China was performed on purified DNA using the Illumina Hiseq PE150 technique by Novogene (Beijing, China), and the genome sequences were assembled by SOAP denovo (v2.04). CISA and Gapclose (v1.12) were used to integrate and refine the assembly. The reads from the SRA database of isolates were assembled using SKESA (v.2.3.0). The quality of the assemblies was evaluated using QUAST (v5.0.2), with an average N50 of 314,661 bp across the genomes. All the genomes were annotated using Prokka (v1.14.6) (Seemann, 2014).

### Core and pan-genome analysis

The pan-genome analysis was applied with Roary (v3.13.0) (Page et al., 2015), identifying the core and accessory genes of ST9 *L. monocytogenes*. The core genes of the 207 isolates were extracted by Snippy with the reference genome of strain EGD-e (NC_003210.1). The phylogenetic tree was constructed based on core genes by MEGA (v7.0.21) with default parameters, using the Maximum Likelihood model, and the bootstraps set up 1,000 replicates. The variation trend of the pan-genome was implied with the script called create_pan_genome_plots.R (https://github.com/sanger-pathogens/Roary/blob/master/bin/create_pan_genome_plots.R).

### Putative plasmid identification and reconstruction

The putative plasmid reconstruction of the isolates from the clean data and the draft assemblies was done by the bioinformatics tools plasmidSPAdes (v3.14.0) (Antipov et al., 2016) and MOB-suite (v3.0.1) (Robertson and Nash, 2018), respectively. Next, the putative plasmids were blasted with the plasmid database of NCBI by the BLASTn algorithm, and the alignments of plasmids with similar sizes were performed by MAUVE (v20150226). Then, the replicon genes of putative plasmid were determined by ABRicate (v1.0.1) with PlasmidFinder database (Carattoli et al., 2014). We believe that the plasmid has two replicon genes since these genes were located in one contig sequence. Two different structures of plasmid replication protein RepA (accession number: AOA49245.1, ZP_00231652.1) representing group 1 (pLM33) and group 2 (pLM80) plasmid (Kuenne et al., 2010), and the amino acid sequences of which were compared against every plasmid sequence using BLASTp for confirmation of putative plasmid group. The putative plasmid genomes were annotated by PROKKA (v1.14.6). The accessory_binary_genes tree of the putative plasmid was created by Roary (v3.13.0) (Page et al., 2015).

### Prophage identification

Prophages were identified by PHASTER with the default parameters (http://phaster.ca) and classified into incomplete, questionable, or complete types according to the completeness score. The putative prophages that did not have special inserted sites were excluded from our analysis. By comparing with the reference genome of ST9 (SLCC2479, NC_018589.1), the chromosomal loci inserted by prophages were identified using MAUVE (v20150226). The intact *comK* (606 nt, AF191725.1) was searched in each isolate using BLASTn. The presence of prophages was regarded as positive if there were two matches (191 nt and 425 nt) of *comK* in the genome (Harrand et al., 2020).

### Identification of virulence genes

For virulence genes identification, genomes of all isolates were analyzed on the website of the Center for Genomic Epidemiology (https://cge.food.dtu.dk/services/VirulenceFinder/), with a minimum of 80% coverage and 90% identity. To determine the presence of truncations in *inlA* coding the internalin A, the full-length InlA protein (800 aa) from the reference isolate (EGD-e, NC_003210.1) was used to create a local database with Diamond (v0.9.32), and every genome was blasted against with the database using BLASTx. The presence of an asterisk (^*^) in the matched query sequences indicated a premature stop codon (PMSC) happening in the genome assembly sequences. The mutation sites were identified by BLASTN with the *inlA* nucleic sequence of the reference isolates (EGD-e, NC_003210.1), and the types of the premature stop codon (PMSC) that were referred to in the previous studies (Van Stelten et al., 2010).

### The resistance-related genes

The antimicrobial-resistant genes of the genomes were identified locally using the ABRicate pipeline (v1.0.0) by the Resfinder database with the default parameter. The presence of 50 genes previously identified as associating with stress resistance, biofilm formation, and resistant islands (Supplementary Table S2) was investigated using the BLASTN algorithm with ≥85% nucleic acid sequence identity and e-value cut-off set at 1 × e^−10^ (Pasquali et al., 2018; Camargo et al., 2019).

## Results

### Single nucleotide polymorphism (SNP) and phylogenetic analysis

The pan-genome analysis was performed on the 207 genomes. The total of 5,330 genes consisted of 2,668 core genes (50.06, 99% ≤ isolates ≤ 100%) and 2,662 accessory genes (49.94%). Accessory gene clusters were classified into three groups: 48 softcore genes (95% ≤ isolates < 99%), 542 shell genes (15% ≤ isolates < 95%) and 2,072 cloud genes (0% ≤ isolates < 15%). The core genes were constant when the number of isolates was more than 25, but the pan genes of ST9 *L. monocytogenes* were rapidly increasing (Supplementary Figure S1).

**Figure 1:**
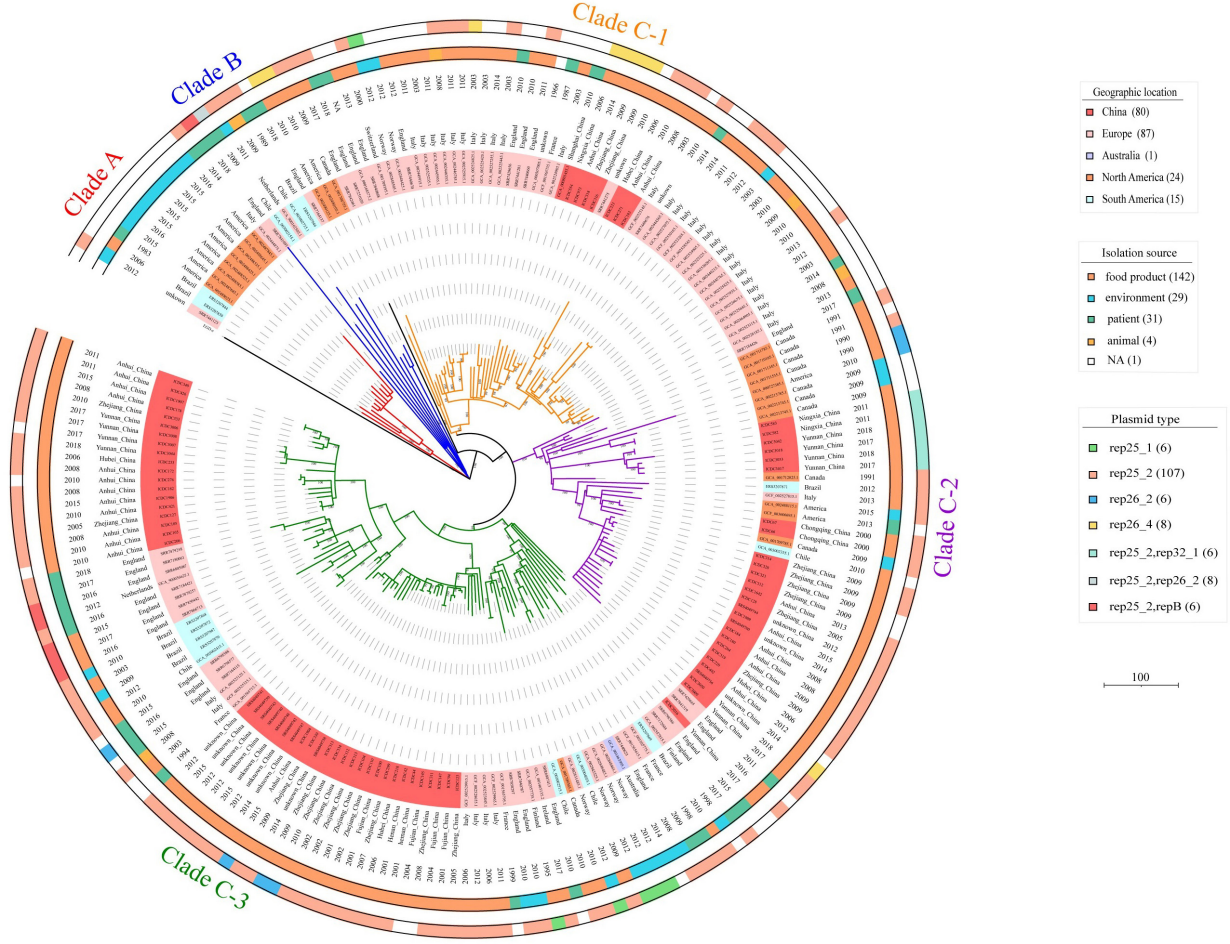
Phylogenetic tree of the 207 ST9 isolates in this study. The tree based on core SNPs was constructed by MEGA, with 1,000 bootstrap replicates. Branches are colored by phylogenetic Clade (Clade A, red; Clade B, blue; Clade C-1, orange; Clade C-2, purple; Clade C-3, green). The geographic location, date of isolation, isolation source, and the plasmid type of the isolates are shown on the tree (from inner to outer circles), according to the color legend shown on the right.

By analyzing the single-nucleotide polymorphism (SNP) of the core genome, the 207 ST9 isolates were classified into three genetic clusters (Clade A, B, and C), and every clade consisted of isolates from different countries and different sources (food, environment, animal, and human) (Figure 1). Most of the ST9 isolates (89.86%, 186/207) belonged to Clade C, which was further divided into three sub-clades (Clade C-1, C-2, and C-3) with isolates of China and other countries. The isolates from North America, South America, and Europe belonged to Clade A and Clade B. However, we found that nine isolates from Brazil belong to Clade A (2), Clade B (1), Clade C-2 (1), and Clade C-3 (5). Notably, most isolates from the same geographic location tended to be clustered together, such as the Chinese ST9 isolates from different provinces and collection times were nearly clustered at the same branches.

### The putative plasmids in ST9 isolates

In this study, 64.25% (133/207) and 3.38% (7/207) of isolates harbored one and two putative plasmids, respectively. After plasmid reconstruction, 147 putative plasmids with sizes ranging from 4.3 to 110 kb were found, including six intact public plasmids (pMF6172, pMF2626, pLM58, pMF4624, pMF4697, and pMF4545). By analyzing the amino acid sequences of RepA which was used to subtype the plasmids of *L. monocytogenes* into two groups (Kuenne et al., 2010), 85.03% (125 /147) and 10.88% (16/147) of the putative plasmids were confirmed to group 1 and group 2. However, 4.08% (6/147) of the putative plasmids carrying the replicon protein RepB instead of RepA, were not classified. Against the PlasmidFinder database, five replicon types of putative plasmid were identified (rep25_1, rep25_2, rep26_2, rep26_4, and rep32_1); a putative plasmid may carry more than one replicon type. Notably, the genes carried by putative plasmids in the same group varied greatly, which is consistent with the previous reports (Hingston et al., 2019). And the isolates belonging to the same clade harbored putative plasmids with different replicon types in the phylogenetic tree (Figure 1).

**Figure 2:**
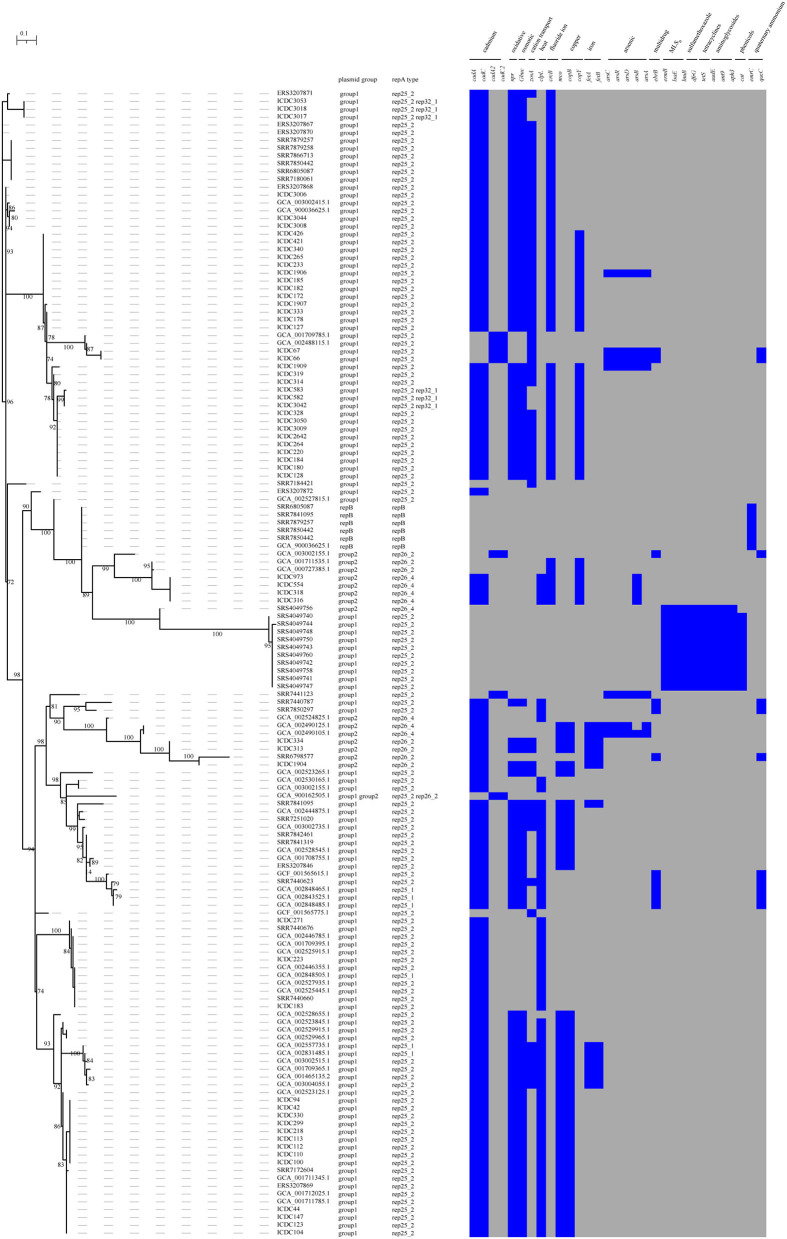
The presence and absence of accessory genes tree of the plasmids. The accessory_binary_genes tree of the putative plasmid was created by Roary. Heatmap showing the presence or absence of the stress resistance genes in the plasmids. Presence and absence genes are marked in blue and gray respectively.

Most plasmids are known to carry some environment-resistance genes. In this study, 80.27% (118/147) of the putative plasmids carried cadmium resistance genes (*cadA* and *cadC*), 63.95% (94/147) carried the genes involving oxidative stress (*npr*) and osmotic stress (*gbuc*) and 42.86% (63/147) harbored heat resistance gene (*clpL*). Other resistance genes encoding cation transport (*zosA*), fluoride ion transporter (*crcB*), copper resistance (*mco* and *copB*), iron resistance (*fetA* and *fetB*), arsenic resistance (*arsC, arcD, arcR, arsB*, and *arsA*), cadmium resistance (*cadA2* and *cadC2)*, and quaternary ammonium compound (*qucC* and *emrC*) existed in 4.8–41.50% of the putative plasmids. In addition, 7.5% (11/147) of the putative plasmids harbored the antibiotic resistance genes for MLSB (*ermB, lnuB*, and *lsaE*), sulfamethoxazole (*dfrG*), aminoglycosides (*ant, aph* and *aadE*), and tetracyclines (*tetS*). About 6.8% (10/147) of putative plasmids also carried the gene *cat* for phenicols resistance (Figure 2).

Nine putative plasmids of ST9 isolates showed high identity with the plasmid of other ST isolates. Six putative plasmids (about 4.3kb) showed high identity (ANI = 100%; coverage > 99.9%) with the plasmid pLmN12-0935 (CP038643.1), belonging to pLMST6 detected in ST6, ST9, ST8, ST31, and ST403 isolates (Kropac et al., 2019). Two putative plasmids (about 91 kb) were closely related (ANI = 100%; coverage = 100%) to plasmid pLM1686 (NZ_MK134858.1) of ST87 isolates (Wang et al., 2019), and one putative plasmid (about 86.6 kb) was similar to plasmid pLMR479a (NZ_HG813248.1) (ANI = 100%; coverage > 99.9%) of ST8 isolates (Naditz et al., 2019).

### Prophages in the genome of *L. monocytogenes* ST9 isolates

A total of 59 putative prophages (17 intact, 21 questionable, and 21 incomplete) were characterized in the completed genomes of 13 isolates, 52 integrated into chromosomes, and seven prophages located on the plasmids (Supplementary Table S4). The putative prophages were also searched among the 194 draft genomes. The virulence genes were not found in the putative prophages according to the database of the VirulenceFinder (https://bitbucket.org/genomicepidemiology/virulencefinder_db/src/master/). Among the stress-resistance genes (Supplementary Table S2), cadmium-resistant genes (*cadA1C1*) were found in 14 putative incomplete prophages with three located on plasmids.

Ten specific prophage insertion sites were identified among the 207 isolates; their locations are shown in Table 1 in comparison with the genome of strain SLCC2479. Prophage ϕP1 (10.7 kb) encoding 17 CDSs has been reported earlier as a cryptic prophage coding monocin and conserved among lineages I and II of *L. monocytogenes* (Wang et al., 2019). A novel prophage ϕP2 (38 kbp) was conserved in 31.88% (66/207) of ST9 isolates in this study (Supplementary Table S3) and inserted into the locus between the gene *fosX* (LMOSLCC2479_RS08965) encoding fosfomycin-resistant thiol transferase (FosX) and the gene *rlmD* (LMOSLCC2479_RS08970) encoding 23S rRNA [uracil (1939)-C(5)]-methyltransferase (RlmD). Prophage ϕP3 was found in isolates ICDC66 and ICDC67 and inserted in the locus (LMOSLCC2479_RS06640) encoding helix-turn-helix transcriptional regulator protein. Prophage ϕP4 was only found in strain ICDC123, which integrated upstream of gene *tsf* (LMOSLCC2479_RS08620) encoding the elongation factor EF-Ts. Other five putative prophages were inserted in the locus adjacent to *tRNA* genes, including *tRNA-Ser* (CGA, LMOSLCC2479_RS03270), *tRNA-Arg* (TCT, LMOSLCC2479_RS06220), *tRNA-Arg* (CCG, LMOSLCC2479_RS12925), *tRNA-Leu* (GAG, LMOSLCC2479_RS10900), and *tRNA-Thr* (GGT, LMOSLCC2479_RS13550). The prophages inserted in gene *comK* were identified in 84.06% (174/207) of ST9 isolates with diverse sequences. Of note, 31 unique prophage profiles (PP: a unique combination of prophages in a genome) were identified based on the prophage insertion sites. The PP1 including ϕP1, ϕtRNA-Arg (TCT), and ϕcomK existed in 48 ST9 isolates, the PP2 including ϕP1 and ϕcomK was found in 32 isolates. However, there were no significant clusters for the distribution of prophage profiles (PP) in the phylogenetic tree of the isolates (Table 2).

**Table 1:** The profiles of prophage insertion sites in 207 isolates of ST9 *L. monocytogenes*.

**Prophage profiles type (PP)**	**The insertion sites of prophage**									**The number of isolates**	**The phylogenetic tree locations of isolates**
	***comk***	***rlmD***	**Gene for helix-turn-helix transcriptional regulator protein**	***tsf***	***tRNA-Arg***	***tRNA-ser***	***tRNA-Arg***	***tRNA-Leu***	***tRNA-Thr***		
1	√				√					48	CladeC-3(20), CladeC-2(14), CladeC-1(10), CladeA(2), CladeB(2)
2	√									32	CladeC-3(22), CladeC-2(5), CladeC-1(5)
3	√	√								26	CladeC-3(17), CladeC-1(9)
4	√	√			√					24	CladeC-3(18), CladeC-1(5), CladeB(1)
5	√				√	√				13	CladeC-3(2), CladeC-2(8), CladeC-1(1), CladeB(2)
6	√					√				10	CladeC-2(10)
7		√								5	CladeC-3(3), CladeC-1(2)
8					√					4	CladeC-1(2), CladeC-2(2)
9										4	CladeC-1(2), CladeA(1), CladeB(1)
10	√	√				√				4	CladeC-3(4)
11						√	√			4	CladeA3(4)
12		√			√					3	CladeC-3(1), CladeC-1(2)
13						√				3	CladeA(3)
14					√	√				3	CladeC-2(1), CladeC-1(1), CladeA(1)
15	√		√		√	√				2	CladeC-2(2)
16	√						√			2	CladeC-3(1), CladeC-1(1)
17					√	√	√			2	CladeA(1), CladeB(1)
18							√	√		2	CladeC-1(2)
19	√	√					√			2	CladeC-3(1), CladeC-1(1)
20		√			√	√				2	CladeC-3(1), CladeC-1(1)
21	√				√		√			2	CladeC-1(1), CladeB(1)
22						√		√		1	CladeC-1(1)
23	√					√	√			1	CladeC-1(1)
24	√							√		1	CladeB(1)
25	√					√				1	CladeC-3(1)
26		√			√				√	1	CladeC-1(1)
27							√			1	CladeC-1(1)
28					√				√	1	CladeC-3(1)
29						√		√		1	CladeC-1(1)
30								√		1	CladeC-1(1)
31	√	√		√						1	CladeC-3(1)

√: The site was inserted by a prophage.

**Table 2:** Premature stop codons (PMSCs) identified in *inlA* of ST9 isolates in this study.

**Mutation type**	**Length(aa)**	**Mutation site**	**References**	**No. (%) of human and animal isolates**	**No. (%) of food and environment isolates**
–	800	–	Glaser et al., 2001	14 (31.82)	25 (14.79)
4	9	6 (deletion A)	Van Stelten et al., 2010	2 (5.88)	37 (21.89)
8	460	1380 (G to A)	Van Stelten et al., 2010	1 (2.94)	30 (17.75)
11	685	2054 (G to A)	Van Stelten et al., 2010	6 (17.65)	17 (10.06)
13	527	1579 (A to T)	Van Stelten et al., 2010	1 (2.94)	1 (0.59)
14	538	1615 (C to T)	Van Stelten et al., 2010	2 (5.88)	1 (0.59)
12	577	1636 (deletion A)	Van Stelten et al., 2010	8 (23.53)	39 (23.07)
19	326	976 (G to T)	Gelbíčová et al., 2015	0 (0)	19 (11.24)
				34 (100)	169 (100)

### Virulence genes and mutation of *inlA* in ST9 isolates

Listeria pathogenicity island (LIPI)-1(*actA, hly, mpl, plcA, plcB*, and *prfA*) and the majority of other 69 virulence genes were conserved in ST9 isolates. But the genes *ami* (invasion)*, inlJ* (internalization), *lisR* (regulation of transcription and translation), *rli55* (regulation of transcription and translation), and *murA* (peptidoglycan modification) were absent in most ST9 isolates, and all isolates did not harbor the Listeria pathogenicity island LIPI-2, LIPI-3, and LIPI-4 (Supplementary Figure S2). Internalin A (InlA) encoded by gene *inlA* plays a key role in the invasion of LM. Most of the ST9 isolates (80.88%, 165/204) had premature stop codon (PMSCs) in gene *inlA* and produced a truncated form of internalin A, and the incidence was higher in the isolates from food and environment (85.21%) than that from humans and animals (68.18%). Seven *inlA* mutation types (type 4, 8, 11, 13, 14, 12 and 19) identified in previous reports (Glaser et al., 2001; Van Stelten et al., 2010) were also found in this study (Table 2).

### The resistance-associated genes in ST9 isolates

**Figure 3:**
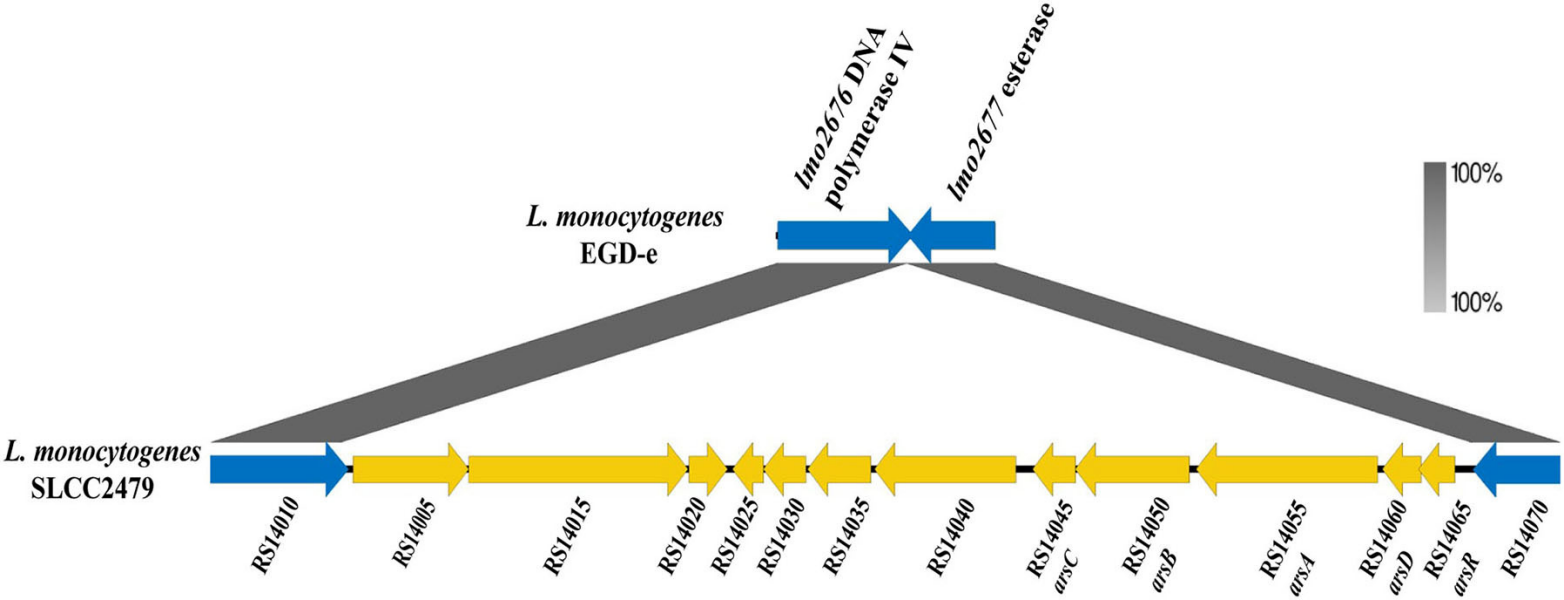
Genomic components of the Tn554-like element of strain SLCC2479. The genes within Tn554-like elements are shown in yellow, and flanked genes of the Tn554-like element in strain EGD-e are shown by blue arrows. The annotation and locus tag of the genes refer to NCBI GenBank (NC_018589.1).

Two putative operons associated with arsenic-resistant *L. monocytogenes* were identified in ST9 isolates. All isolates of Clade A harboring LGI2 carried an arsenic resistance cassette (*arsD1A1R1D2R2A2B1B2*), which was identical to the previous report by Lee et al. (2017) (Supplementary Figure S4). A Tn554-like element was identified in 78.26% (162/207) ST9 isolates, which carried an arsenic-resistant operon (*arsCBADR*) and showed 70% amino acid identity with that of *Enterococcus faecalis* (Figure 3) (Kuenne et al., 2013). It mostly (95.68%, 155/162) inserted into the similar locus between the genes *lmo2676* (DNA polymerase IV) and *lmo2677* (esterase) on the chromosome of strain EGD-e. Interestingly, the Tn554-like elements were found in the plasmids of seven ST9 isolates from America (2), Europe (1), and China (4).

Genes associated with adaptation to cold (*cspB, cspD, lmo0866, lmo1722, lmo1378*, and *lmo0288*), low pH (*lmo2434, lmo2363, lmo0043, lmo0796, lmo2362, lmo2391, lmo0039, lmo0037, lmo0913, lmo0036*, and *lmo1367*), high salt concentration (*lmo2748, lmo2092, lmo1014, lmo1015, lmo1016, lmo1428, lmo1427, lmo1426*, and *lmo1425*), and biofilm formation (*lmo0673, lmo0435, lmo1460, lmo2504, lmo1288*, and *inlL*), and SSI-1 containing five genes (*lmo0444–lmo0448*) to resist acidic, bile, gastric, and salt stresses were conserved (identity > 99%) in all ST9 isolates (100%, 207/207) (Supplementary Figure S3) (Pasquali et al., 2018). But none of the isolates had SSI-2, which is involved in the alkaline and oxidative stress response (Harter et al., 2017).

The Benzalkonium Chloride (BC) tolerance-related genes (*bcrABC, qacH, emrC, emrE*, and *quaC*) were screened in ST9 isolates. The *bcrABC* cassette was prevalent and existed in 7.73% (16/207) of the isolates in Clade C-2, C-3, and Clade B, followed by the transposons Tn6188 carrying genes *tnpABC, tetR*, and *qacH* in isolates of Clade C-1 (5.31%, 11/207). The gene *emrC* of the plasmid pLMST6 was found in 2.90% (6/207) of the isolates in Clade B and Clade C-3, the gene *emrE* was identified in one isolate, but the gene *quaC* was absent in all ST9 isolates.

All the ST9 isolates harbored the fosfomycin-resistant related gene *fosX*, and 11 isolates from China carried plasmids with antibiotic genes detailed in the plasmids part, and three isolates from Europe harbored the tetracycline resistance gene *tetM* on the chromosome (Supplementary Figure S4).

## Discussion

Previous studies have reported the various ecological niche adaptation and pathogenic potential of different *L. monocytogenes* CCs isolates (Maury et al., 2016, 2019). As the common clone of *L. monocytogenes* worldwide, CC9 isolates were highly associated with meat products and meat-processing environments (Chenal-Francisque et al., 2011; Fagerlund et al., 2020). The ST9 isolate could outcompete other types of isolates when growing in a meat-based growth medium (BHI) in the presence of *Listeria innocua* (Heir et al., 2018). However, the mechanism of adaption to the meat environment was not clear for ST9 isolates. In this study, WGS was employed to investigate the population structure and genetic features of 207 ST9 *L. monocytogenes* isolates from various sources and different regions in the world.

The pan-genome of ST9 *L. monocytogenes* was increasing with the number of isolates and indicates the open-genome of ST9 isolates, which is larger than that of ST87 isolates (3,687 genes, obtained by CD-HIT) and closer to that of the *Listeria* species (5,469 genes) (Kuenne et al., 2013; Wang et al., 2019), but smaller than that of ST155 isolates (6,422 genes; Wagner et al., 2020). The variation trend of pan-core genes indicates that ST9 isolates have a stable open genome and permit the integration of foreign DNA (Kuenne et al., 2013). Since 2000, CC9 isolates have occurred in high frequency and become a predominant clone worldwide (Bergholz et al., 2018). This suggests that the ST9 LM, with its increasing ability to adapt to new niches, is in a stage of rapid evolvement and spread.

Based on the SNPs analysis of the core genome, all ST9 isolates from 14 countries were divided into three clades, namely Clade A, B, and C, with Clade C being dominant. The isolates from the same counties were distributed into different clades, indicating the high genomic diversity of ST9 strains, which is consistent with previous studies (Camargo et al., 2019). The isolates from different countries crossly distributed in Clade C-3 suggest ST9 *L. monocytogenes* has spread between different countries. All the Chinese isolates from various collection times and geographic locations clustered in Clade C suggesting the same original clone strains have spread to different provinces through food contamination or other ways in China. The clinical isolates existing at different branches showed the potential pathogenicity of ST9 isolates in humans (Figure 1).

As a cell-wall protein binding to E-cadherin on the host epithelial cell, InlA is associated with passing through the intestinal barrier (Su et al., 2019). A total of 32 mutation types of the *inlA* gene have been identified (Van Stelten et al., 2010; Gelbíčová et al., 2015; Toledo et al., 2018), including seven types identified in this study, and InlA with type 11, 13, and 19 PMSCs were not functional (Toledo et al., 2018). Attenuated invasion in human intestinal epithelial cells and a guinea pig model was observed for the isolates with truncated InlA (Nightingale et al., 2008; Kanki et al., 2015). PMSC mutations in *inlA* occurred in 80.88% of ST9 isolates in this study, and 93.48 and 100% of 1/2c isolates in previous reports (Kanki et al., 2015; Su et al., 2019). In addition, 51.61% (16/31) of clinical ST9 isolates carried the truncated InlA, which suggests the probable synergetic effect of other virulent factors.

Some studies proved the contribution of plasmids in *L. monocytogenes* to stress response (Pöntinen et al., 2017; Naditz et al., 2019). It was proposed that 28–90% of *L. monocytogenes* carried the plasmid depending on the STs (Naditz, 2020), and 67.14% (139/207) of ST9 isolates harbored putative plasmid in this study; the frequency is higher than that of other STs isolates such as ST155 and ST87 (Wang et al., 2019; Wagner et al., 2020). The plasmids were genetically conserved in ST121 and ST87 isolates (Schmitz-Esser et al., 2015; Wang et al., 2019), but were variable in size and genome structure in the ST9 isolates, which is consistent with that of previous reports (Naditz, 2020). Meanwhile, the genes of putative plasmids belonging to the same group were obviously diverse. The putative plasmids of ST9 isolates harbored some resistance-related genes with high variety, such as *gbuC* and *clpL*, and some metal resistance-related genes, which would increase the adaption ability of ST9 isolates to various adverse conditions in food or food-processing environments. Furthermore, six putative plasmids showed high identity with plasmid pMLST6, which harbored genes encoding multidrug efflux pump protein (EmrC) and was related to unfavorable disease outcome (Kropac et al., 2019), suggesting that these plasmids probably increase the virulence of ST9 isolates, which need to be confirmed.

The prophages are also generally associated with the environmental adaptation of isolates, including biofilm formation, osmotic resistance, acid resistance, and rapid niche adaptation (Wang et al., 2010; Verghese et al., 2011; Vu et al., 2019). Similar to other STs isolates of LM, the prophages of ST9 isolates were mostly inserted in the sites adjacent to the *tRNA* locus in the genome (Schmitz-Esser et al., 2015; Fox et al., 2016; Wang et al., 2019). Prophage ϕcomK are commonly considered as active regulatory switches in enhancing niche adaptation, biofilm formation, and attachment capability (Verghese et al., 2011), and would give ST9 isolates the advantages of survival and reproduction. In addition, prophage ϕP2, which was conserved in 31.88% of ST9 isolates of Clade C-1 and Clade C-3, is one of the important drivers of short-term genome evolution for ST9 isolates.

LGI2 carrying *arsD1A1R1D2R2A2B1B2* cassette (arsenic resistance), which was initially identified in CC2 strain Scott A (Briers et al., 2011) and ST9 isolates of Clade A, might contribute to the enhanced virulence of isolates with other determinants (Lee et al., 2017). The exact function of LGI2 in ST9 isolates needs to be confirmed by further studies. The arsenic resistance cassette (*arsCBADR*) harbored on a Tn554-like element was first identified in the serotype 1/2c strain SLCC 2372 (Kuenne et al., 2013). It was expressed in the plasmid of seven ST9 isolates in this study, suggesting it was specific to the CC9 clone of *L. monocytogenes* and arsenic resistance could be acquired through plasmid horizontal transfer. These arsenic detoxification resistance-related genes also had additional cellular functions or influenced the environmental fitness of ST9 isolates (Lee et al., 2017; Parsons et al., 2020).

Some resistance genes that are associated with adaptation to cold, low pH, high salt concentration, and biofilm formation were conserved in ST9 *L. monocytogenes*, which could benefit the isolates widely surviving in the environment. The BC resistance genes (*bcrABC* and *qacH*) were found in some ST9 isolates from food or food-processing environment and their role was confirmed (Maury et al., 2019). ST9 was reported as the predominant sequence type (24.4%) in BC resistance isolates from Swiss and Finnish samples (Meier et al., 2017), but fewer isolates (16.42%, 34/207) were found harboring the BC resistance genes in this study, potentially due to the different environment and exposure to disinfectants with quaternary ammonia compounds (QAC). Though the antimicrobial resistance of *L. monocytogenes* is not challenging, some resistant isolates are being increasingly reported in recent years (Wilson et al., 2018). Eleven multiple drug-resistant ST9 isolates of 28 resistant isolates were reported from 2,862 *L. monocytogenes* in China (Yan et al., 2019). Antimicrobial resistance genes of 11 ST9 isolates were carried on plasmids in this study, showing the possibility of horizontal transfer of antibiotic-resistant genes.

About the limitations of this study, because the high-quality genome sequences of the ST9 strains from some other representative countries or regions with detailed sources could not be found in public databases currently, more possible genetic diversity of ST9 strains could not be revealed in this study. In addition, other possible virulence-related factors of some clinical ST9 strains carried the truncated InlA, which needs to be confirmed in further research.

## In conclusion

In this study, the population structure and genetic features of *L. monocytogenes* ST9 isolates worldwide were investigated by WGS analysis. A total of 147 putative plasmids with variant sizes and genome structure, ten types of insertion sites of prophage, stress resistance genes, Tn554-like element, and PMSC in *inlA* with seven mutation types were found in ST9 isolates. These genetic elements can play an important role in genomic diversity, growth in suboptimal conditions, and the virulence variation of *L. monocytogenes* ST9 isolates. These findings would be helpful for making effective strategies to control and prevent foodborne listeriosis caused by ST9 *L. monocytogenes*.

## Data availability statement

The datasets presented in this study are deposited in the China National Microbiology Data Center (NMDC), with accession number NMDC60016796 to NMDC60016674 (https://nmdc.cn/resource/genomics/genome/).

## Author contributions

ZS, SJ, and CY conceived and designed the research. YiW, PM, LLi, and HJ isolated and extracting the genome of ST9 isolates used. ZS, SJ, YaW, and LLu contributed for raw data analysis and bioinformatics analysis. ZS, YaW, and CY wrote and revised the paper. All authors read and approved the final manuscript.

## Funding

This work was supported by grants from the National Institute for Communicable Disease Control and Prevention, China CDC (2021ZZKT003 and 131031102000210003-07007), the National Key R&D Program of China (2018YFC1603800), and the National Natural Science Foundation of China (31800004).

## Conflict of interest

The authors declare that the research was conducted in the absence of any commercial or financial relationships that could be construed as a potential conflict of interest.

## Publisher's note

All claims expressed in this article are solely those of the authors and do not necessarily represent those of their affiliated organizations, or those of the publisher, the editors and the reviewers. Any product that may be evaluated in this article, or claim that may be made by its manufacturer, is not guaranteed or endorsed by the publisher.

## Supplementary material

Supplementary Figure S1 The pan-genome varies as ST9 genomes are added in this study. It was implied with the script called *create_pan_genome_plots.R* (https://github.com/sanger-pathogens/Roary/blob/master/bin/create_pan_genome_plots.R).

Supplementary Figure S2 Virulence gene profiles of 207 isolates in this study. The phylogenetic tree was constructed by MEGA. The gene's presence or absence was shown by blue and gray, and the color was changed according to the identity. From left to right, genes consist of LIPI-1 (*prfA, plcA, plcB, hly, mpl*, and *atcA*), LIPI-3 (*llsAGHXYDP*), LIPI-4 (*Clip80459_02324 to Clip80459_02329*) and other virulence genes about internalization (*inlABCFHJK*), adherence (*ami, dltA, fbpA, lap*, and *lapB*), invasion (*aut, iap, ipeA, recA*, and *vip*), intracellular survival (*clpB, clpc, clpe, clpp, dal, fri, htrA, lplA1, oppA, perR, prsA2, pycA, relA, sipZ, sod, svpA, tig*, and *uHpt*), regulation of transcription and translation (*agrA, ctsR, gmar, fur, hfq, lhrC, lisK, lisR, mogR, rli55, rli60, rsbv, sigB, stp*, and *virR*), surface protein anchoring (*lgt, lsp, secA2, sipX, srtA*, and *srtB*), peptidoglycan modification (*degU, murA, oatA*, and *pgdA*), immune modulation (*chiA, lipA, intA, pgl*, and *tcsA*), bile-resistance (*bsh, bilE*), teichoic acid biosynthesis (*gtcA*), motility (*flaA, flgC*, and *flgE*), membrane integrity (*ctaP, mprF*), and metabolic regulator (*codY*) are shown.

Supplementary Figure S3 Profiles of environment adaption genes of 207 isolates in this study. The gene's presence or absence was shown by blue and gray, and the color was changed according to the identity. From left to right, the genes involving adaptation to low pH (*lmo2434, lmo2363, lmo0043, lmo0796, lmo2362, lmo2391, lmo0039, lmo0037, lmo0913, lmo0036*, and *lmo1367*), biofilm (*lmo0673, lmo0435, lmo1460, lmo2504, lmo1288*, and *inlL*), desiccation (*lmo0676, lmo0679, lmo0696, lmo0706, lmo0686, lmo0699*, and *lmo0693*), cold adaptation (*cspB, cspD, lmo0866, lmo1722, lmo1378*, and *lmo0288*), high salt concentration (*lmo2748, lmo2092, lmo1014, lmo1015, lmo1016, lmo1428, lmo1427, lmo1426*, and *lmo1425*), and SSI-1 (*lmo0444, lmo0445, lmo0446, lmo0447*, and *lmo0448*) are shown.

Supplementary Figure S4 PMSCs in *inlA*, Tn554-like element, prophage ϕcomK, ϕP2, and a few resistant gene profiles of the 207 isolates sequenced in this study. The presence and absence of genes are marked in blue and gray, respectively.

Supplementary Table S1 207 ST9 *L. monocytogenes* genome sequences used in this study.

Supplementary Table S2 The genes associated with stress resistance and resistant island.

Supplementary Table S3 Gene content of prophage ϕP2 of ST9 isolate AT3E. The gene annotation was reported with NCBI annotation (NZ_CP023752.1).

Supplementary Table S4 59 putative prophages were characterized in 13 complete genomes.
