# Supplementary figures and images for: The population structure and genetic diversity of Listeria monocytogenes ST9 strains based on genomic analysis
Source: Front Microbiol. 2022 Nov 8;13:982220. doi: 10.3389/fmicb.2022.982220 (PMC9680904; doi:10.3389/fmicb.2022.982220)

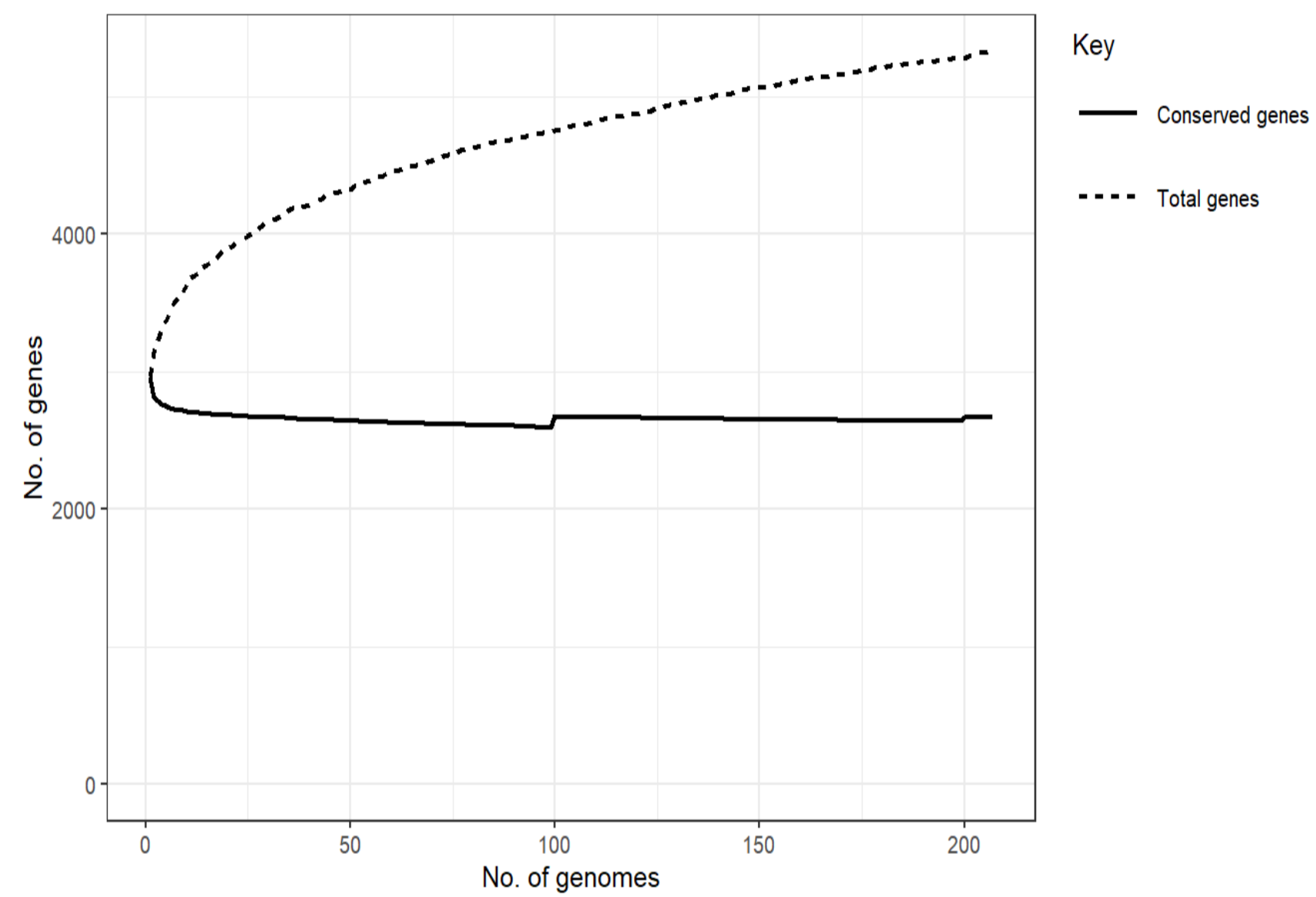

Supplement: Supplementary Figure S1 — The pan-genome varies as ST9 genomes are added in this study. It was implied with the script called create_pan_genome_plots.R (https://github.com/sanger-pathogens/Roary/blob/master/bin/create_pan_genome_plots.R). [file Data_Sheet_1.PDF]

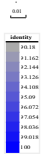

Clade A Clade B

Clade C-1

Clade C-2

Clade C-3

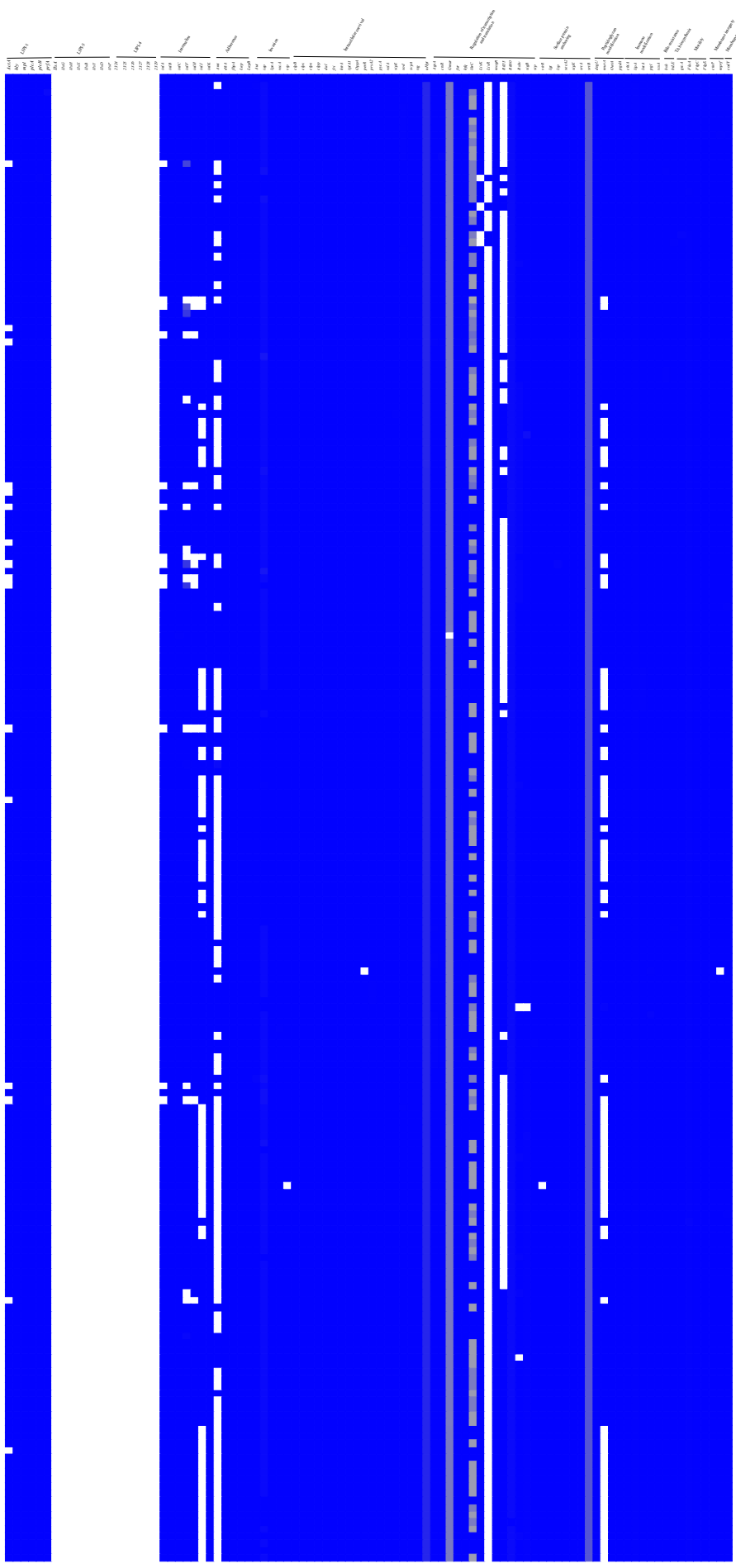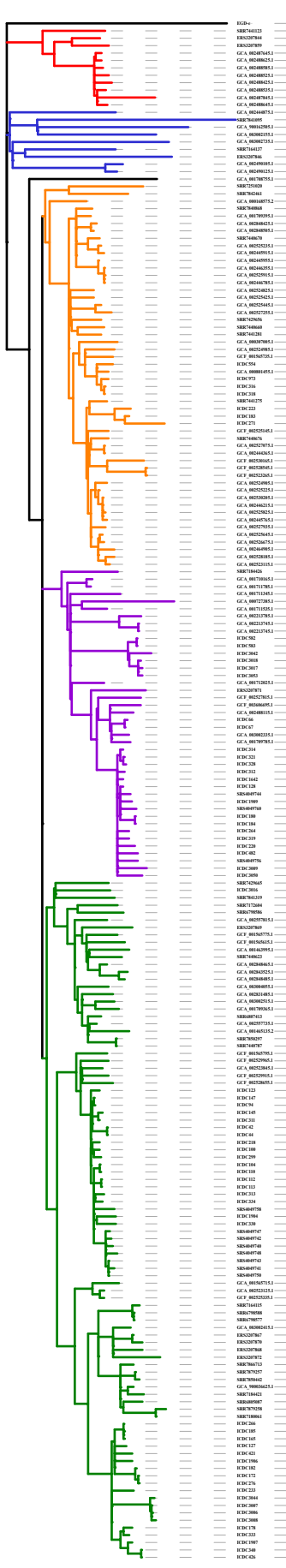

Supplement: Supplementary Figure S2 — Virulence gene profiles of 207 isolates in this study. The phylogenetic tree was constructed by MEGA. The gene's presence or absence was shown by blue and gray, and the color was changed according to the identity. From left to right, genes consist of LIPI-1 (prfA, plcA, plcB, hly, mpl, and atcA), LIPI-3 (llsAGHXYDP), LIPI-4 (Clip80459_02324 to Clip80459_02329) and other virulence genes about internalization (inlABCFHJK), adherence (ami, dltA, fbpA, lap, and lapB), invasion (aut, iap, ipeA, recA, and vip), intracellular survival (clpB, clpc, clpe, clpp, dal, fri, htrA, lplA1, oppA, perR, prsA2, pycA, relA, sipZ, sod, svpA, tig, and uHpt), regulation of transcription and translation (agrA, ctsR, gmar, fur, hfq, lhrC, lisK, lisR, mogR, rli55, rli60, rsbv, sigB, stp, and virR), surface protein anchoring (lgt, lsp, secA2, sipX, srtA, and srtB), peptidoglycan modification (degU, murA, oatA, and pgdA), immune modulation (chiA, lipA, intA, pgl, and tcsA), bile-resistance (bsh, bilE), teichoic acid biosynthesis (gtcA), motility (flaA, flgC, and flgE), membrane integrity (ctaP, mprF), and metabolic regulator (codY) are shown. [file Data_Sheet_2.PDF]
