# Supplementary material for: The population structure and genetic diversity of Listeria monocytogenes ST9 strains based on genomic analysis
Source: Front Microbiol. 2022 Nov 8;13:982220. doi: 10.3389/fmicb.2022.982220 (PMC9680904; doi:10.3389/fmicb.2022.982220)
Supplement: Supplementary Figure S3 — Profiles of environment adaption genes of 207 isolates in this study. The gene's presence or absence was shown by blue and gray, and the color was changed according to the identity. From left to right, the genes involving adaptation to low pH (lmo2434, lmo2363, lmo0043, lmo0796, lmo2362, lmo2391, lmo0039, lmo0037, lmo0913, lmo0036, and lmo1367), biofilm (lmo0673, lmo0435, lmo1460, lmo2504, lmo1288, and inlL), desiccation (lmo0676, lmo0679, lmo0696, lmo0706, lmo0686, lmo0699, and lmo0693), cold adaptation (cspB, cspD, lmo0866, lmo1722, lmo1378, and lmo0288), high salt concentration (lmo2748, lmo2092, lmo1014, lmo1015, lmo1016, lmo1428, lmo1427, lmo1426, and lmo1425), and SSI-1 (lmo0444, lmo0445, lmo0446, lmo0447, and lmo0448) are shown. [file Data_Sheet_3.PDF]

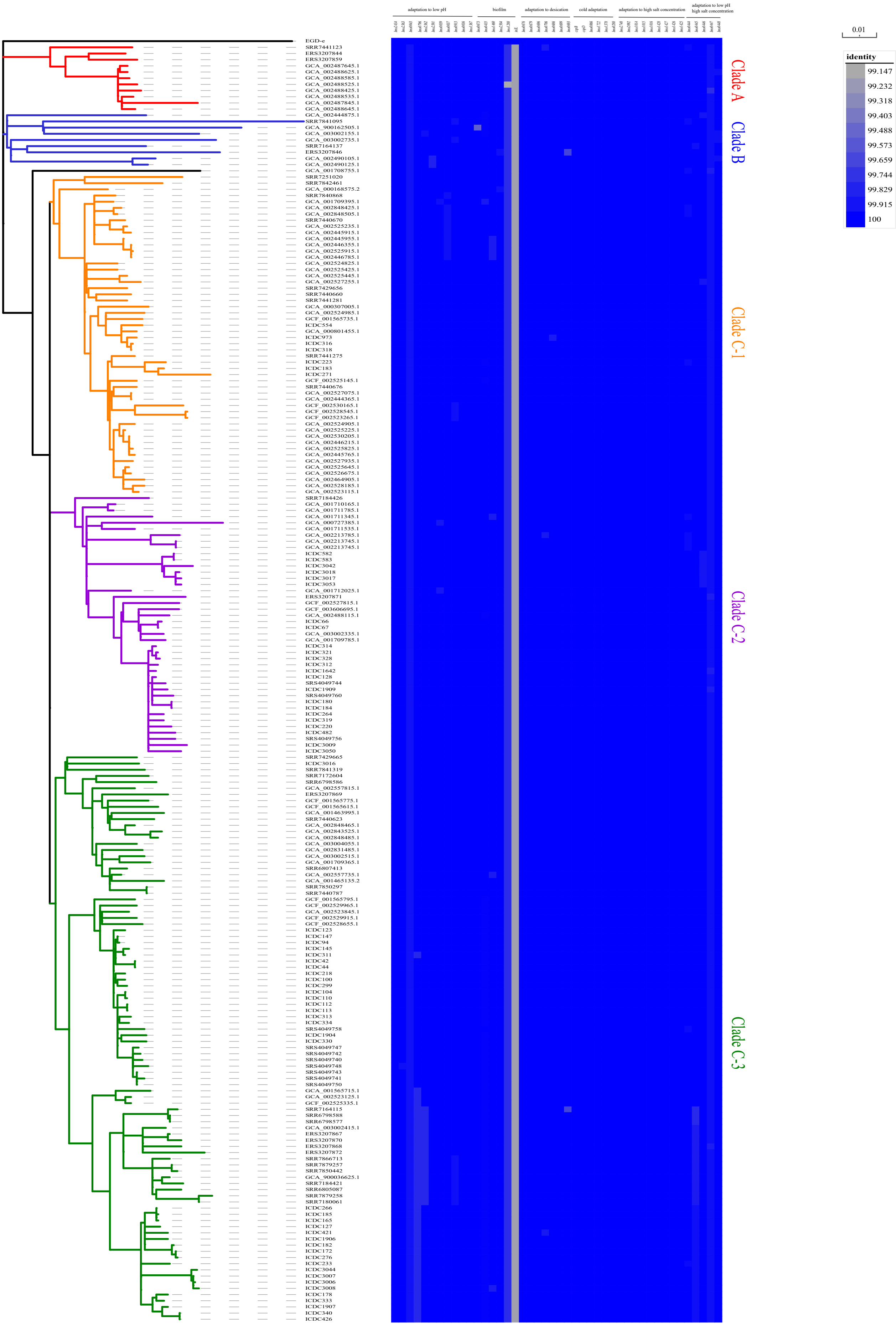

adaptation to low pH
biofilm
adaptation to desication
cold adaptation
adaptation to high salt concentration
adaptation to low pH high salt concentration
0.01
identity
99.147
99.232
99.318
99.403
99.488
99.573
99.659
99.744
99.829
99.915
100
Clade A
Clade B
Clade C-1
Clade C-2
Clade C-3
