# Supplementary material for: The population structure and genetic diversity of Listeria monocytogenes ST9 strains based on genomic analysis
Source: Front Microbiol. 2022 Nov 8;13:982220. doi: 10.3389/fmicb.2022.982220 (PMC9680904; doi:10.3389/fmicb.2022.982220)
Supplement: Supplementary Figure S4 — PMSCs in inlA, Tn554-like element, prophage ϕcomK, ϕP2, and a few resistant gene profiles of the 207 isolates sequenced in this study. The presence and absence of genes are marked in blue and gray, respectively. [file Data_Sheet_4.PDF]

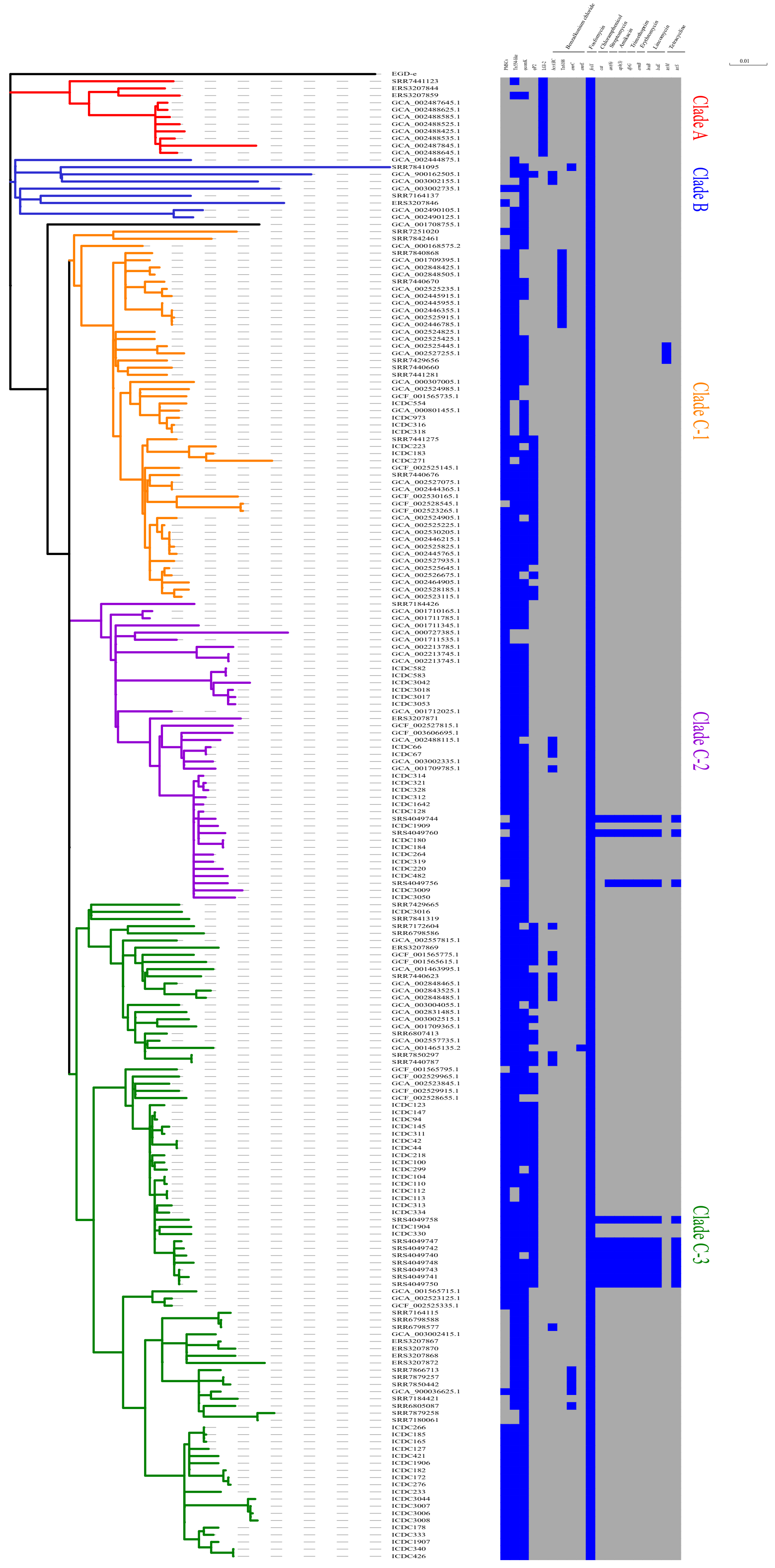

EGD-e
SRR7441123
ERS3207844
ERS3207859
GCA_002487645.1
GCA_002488625.1
GCA_002488585.1
GCA_002488525.1
GCA_002488425.1
GCA_002488535.1
GCA_002487845.1
GCA_002488645.1
GCA_002444875.1
SRR7841095
GCA_900162505.1
GCA_003002155.1
GCA_003002735.1
SRR7164137
ERS3207846
GCA_002490105.1
GCA_002490125.1
GCA_001708755.1
SRR7251020
SRR7842461
GCA_000168575.2
SRR7840868
GCA_001709395.1
GCA_002848425.1
GCA_002848505.1
SRR7440670
GCA_002525235.1
GCA_002445915.1
GCA_002445955.1
GCA_002446355.1
GCA_002525915.1
GCA_002446785.1
GCA_002524825.1
GCA_002525425.1
GCA_002525445.1
GCA_002527255.1
SRR7429656
SRR7440660
SRR7441281
GCA_000307005.1
GCA_002524985.1
GCF_001565735.1
ICDC554
GCA_000801455.1
ICDC973
ICDC316
ICDC318
SRR7441275
ICDC223
ICDC183
ICDC271
GCF_002525145.1
SRR7440676
GCA_002527075.1
GCA_002444365.1
GCF_002530165.1
GCF_002528545.1
GCF_002523265.1
GCA_002524905.1
GCA_002525225.1
GCA_002530205.1
GCA_002446215.1
GCA_002525825.1
GCA_002445765.1
GCA_002527935.1
GCA_002525645.1
GCA_002526675.1
GCA_002464905.1
GCA_002528185.1
GCA_002523115.1
SRR7184426
GCA_001710165.1
GCA_001711785.1
GCA_001711345.1
GCA_000727385.1
GCA_001711535.1
GCA_002213785.1
GCA_002213745.1
GCA_002213745.1
ICDC582
ICDC583
ICDC3042
ICDC3018
ICDC3017
ICDC3053
GCA_001712025.1
ERS3207871
GCF_002527815.1
GCF_003606695.1
GCA_002488115.1
ICDC66
ICDC67
GCA_003002335.1
GCA_001709785.1
ICDC314
ICDC321
ICDC328
ICDC312
ICDC1642
ICDC128
SRS4049744
ICDC1909
SRS4049760
ICDC180
ICDC184
ICDC264
ICDC319
ICDC220
ICDC482
SRS4049756
ICDC3009
ICDC3050
SRR7429665
ICDC3016
SRR7841319
SRR7172604
SRR6798586
GCA_002557815.1
ERS3207869
GCF_001565775.1
GCF_001565615.1
GCA_001463995.1
SRR7440623
GCA_002848465.1
GCA_002843525.1
GCA_002848485.1
GCA_003004055.1
GCA_002831485.1
GCA_003002515.1
GCA_001709365.1
SRR6807413
GCA_002557735.1
GCA_001465135.2
SRR7850297
SRR7440787
GCF_001565795.1
GCF_002529965.1
GCA_002523845.1
GCF_002529915.1
GCF_002528655.1
ICDC123
ICDC147
ICDC94
ICDC145
ICDC311
ICDC42
ICDC44
ICDC218
ICDC100
ICDC299
ICDC104
ICDC110
ICDC112
ICDC113
ICDC313
ICDC334
SRS4049758
ICDC1904
ICDC330
SRS4049747
SRS4049742
SRS4049740
SRS4049748
SRS4049743
SRS4049741
SRS4049750
GCA_001565715.1
GCA_002523125.1
GCF_002525335.1
SRR7164115
SRR6798588
SRR6798577
GCA_003002415.1
ERS3207867
ERS3207870
ERS3207868
ERS3207872
SRR7866713
SRR7879257
SRR7850442
GCA_900036625.1
SRR7184421
SRR6805087
SRR7879258
SRR7180061
ICDC266
ICDC185
ICDC165
ICDC127
ICDC421
ICDC1906
ICDC182
ICDC172
ICDC276
ICDC233
ICDC3044
ICDC3007
ICDC3006
ICDC3008
ICDC178
ICDC333
ICDC1907
ICDC340
ICDC426
PMSCs
Tn554-like
φcomK
φP2
LGI-2
bcrABC
Tn6188
emrC
emrE
fosX
cat
ant(6)
aph(3)
dfrG
ermB
lnuB
lsaE
tetM
tetS
Benzalkonium chloride
Fosfomycin
Chloramphenicol
Streptomycin
Amikacin
Trimethoprim
Erythromycin
Lincomycin
Tetracycline
0.01
Clade A
Clade B
Clade C-1
Clade C-2
Clade C-3
